# Navigating Complexity: Spiritual Care Discourses Among Swedish Palliative Care Professionals

**DOI:** 10.1007/s10943-024-02106-4

**Published:** 2024-08-20

**Authors:** Emma Lundberg, Anneli Ozanne, Lisen Dellenborg, Joakim Öhlén, Daniel Enstedt

**Affiliations:** 1 Institute of Health and Care Sciences, Sahlgrenska Academy, University of Gothenburg, P.O. Box 457, 413 46 Gothenburg, Sweden; 2 Department of Neurology, Sahlgrenska University Hospital, Blå Stråket 7, 413 46 Göteborg, Sweden; 3 Centre for Person-Centred Care (GPCC), University of Gothenburg, Arvid Wallgrens Backe, 413 46 Gothenburg, Sweden; 4 Palliative Centre, Sahlgrenska University Hospital Region Västra Götaland, Lilla Kapplandsgatan 7C, 421 37 Västra Frölunda, Sweden; 5 Department of Literature, History of Ideas, and Religion, University of Gothenburg, Renströmsgatan 6, 412 55 Gothenburg, Sweden

**Keywords:** Spiritual care, Palliative care, Discourse analysis, Religion, Spirituality

## Abstract

Through discourse analysis of focus groups, this study investigates how palliative care professionals in Sweden engage with “spiritual care,” “religion” and “spirituality.” Our results reveal a common assumption that religion is “visible,” but at the same time private. Furthermore, we observed a secular and nonreligious positioning, marked by frequent "us versus them" rhetoric, especially in discussions about truth telling. The findings illustrate a view of belonging to a secular society in which a discourse of static understanding of religion dominated, indicating a vague religious literacy. This study indicates a need among healthcare professionals to discern, understand and relate to non-visible forms of religion.

## Introduction

Patients cared for in palliative care services have the right to care that meets their spiritual and religious needs, a principle highlighted on an international level, and it has been argued that this is a responsibility for healthcare professionals (Ferrell et al., 2018; Puchalski et al., 2009; National Consensus Project for Quality Palliative Care, 2018). Despite this, spiritual and religious needs are deprioritized in health care (e.g., Balboni et al., 2007; Hermann, 2007; Leget, 2017). The neglect of meeting such needs has led to distress, as well as ambiguity in professional responsibilities (Pentaris, 2019). Lack of knowledge, training and skills has been recognized as one reason for this (e.g., Edwards et al., 2010; Lundberg, 2023; Norberg, 2018; van der Weegen et al., 2019), as well as organizational and structural factors (Dellenborg & Enstedt, 2023a, b). Research shows that healthcare professionals wish to give spiritual care at the end of life, but experience difficulties regarding how to do so (Balboni et al., 2014; Henoch et al., 2015; Strang et al., 2002).

Organizations such as WHO (2024) and the European Association of Palliative Care (EAPC) (2023) emphasize the integration of spirituality and religion into palliative care. Religious needs are said to become more prominent when life is at risk (Strang, 2002; Van der Geest, 2005), even for those previously disengaged from spiritual or religious matters (Ammerman, 2013; Zingmark & Granberg-Axèll, 2023). According to Murray et al. (2004, p.40), spiritual needs can be defined as “the needs and expectations which humans have to find meaning, purpose and value in their life. Such needs can be specifically religious, but even people who have no religious faith or are not members of an organized religion have belief systems that give their lives meaning and purpose.”

Spiritual needs are also said to include the loss of roles and self-identity, and fear of dying (Grant et al., 2004). Spiritual and religious needs such as praying, reading and reciting texts and also food preferences frequently emerge in healthcare situations, according to Nordin and Schölin (2011) and Wedel (2011). Both religion and spirituality have been described as essential coping resources during illness and when facing the end of life (Ahmadi, 2006; Büssing et al., 2009; Puchalski, 2008; Thuné-Boyle et al., 2006). A patient’s religious and spiritual needs and considerations are connected to physical and mental well-being (Koenig, 2012; Norberg, 2018; Puchalski, 2001) and quality of life (Balboni et al., 2007).

Sweden is frequently characterized as secular (Willander, 2019; Zuckerman, 2009), with a highly individualized population adopting secular-rational values and self-expression values (Inglehart & Welzel, 2010). A general assumption about society becoming increasingly secularized suggests that people no longer practice religion or interpret life and death through the lens of religion or belief. The assumed correlation between an increased secularization of societal institutions, declining membership in the former state church, and religious activity and beliefs among the Swedish population in general may lead to the faulty conclusion that religion is vanishing on all levels of society.

While societal institutions, such as health care, can nowadays be described as secularized, and the decline of membership in the Church of Sweden is a fact—even though more than 50 percent of the population are still members (Svenska kyrkan, 2022)—the situation in Sweden needs to be further nuanced. Contemporary research on Sweden’s religious landscape emphasizes the presence of religiosity and spirituality in new forms and scholars have argued for a so-called spiritual revolution, in which Christianity and other established religions have given way to a more holistic spirituality (Enstedt & Plank, 2023a, 2023b; Frisk & Åkerbäck, 2013; Heelas & Woodhead, 2005; Hornborg, 2012). At the same time, the religious landscape in Sweden has undergone significant transformation, through waves of migration, to a more diverse, multiethnic and multi-religious composition (Willander, 2019; Andersson & Sander, 2015). In sum, this means that healthcare professionals now regularly encounter and operate in an environment rich in a diversity of religious and spiritual beliefs and practices (Dellenborg & Enstedt, 2023a; b).

## Aim

The aim of this study is to deepen the understanding of how palliative care professionals engage with the concept of spiritual care, as well as associated concepts such as religion and spirituality, and how norms and values related to these concepts are part of, and form, the understanding of care. By identifying challenges and possibilities associated with spiritual care, the study seeks to contribute to increased awareness and competency development in this complex area of palliative care.

## Methods

A qualitative design with focus groups was applied to generate data on palliative care professionals’ experiences and views from different perspectives. We utilized discourse analysis to examine how palliative care professionals perceive and utilize the concept of “spiritual care,” and in which ways “religion,” and “spirituality” may constitute a significant part of the discourse.

### Discourse Analysis

A discourse-theoretical perspective underlines language’s pivotal role in shaping reality through discourses (Fairclough, 2013; Foucault, 2002, 2011). Drawing from Foucault’s theory, it explores how language constructs power dynamics, knowledge and societal realities, emphasizing how discourses express ideologies, norms and power structures. While at times perceived as fluid, discourses manifest themselves tangibly in institutions and practices, engendering resistance to change.

Discourses assign meaning, framing our perception of “reality.” While discourses are intertwined with corporeality, institutional settings and material forms, discourse analysis primarily focuses on understanding the functional aspect of language. It explores how language usage shapes the construction of identities, relationships, beliefs and systems of knowledge (Fairclough, 2013; Hjelm, 2021).

In this study, we use discourse analysis to evaluate the usage by and the meaning of the concept “spiritual care” for palliative care professionals, as well as related concepts such as “spirituality” and “religion.” From a discursive standpoint, these terms were considered contextually dependent and devoid of inherent meaning. Through the use of discourse analysis, we aimed to uncover implicit understandings and prevalent themes associated with the concept of spiritual care and to discern and comprehend the contextual factors that infuse this concept with meaning.

“Spiritual care” derives meaning from its context. Throughout this process, we sought out nodal points (Laclau, 1985). Nodal points serve as central signs that hold a special significance, organizing the other signs around them. The meaning of the other signs is derived from their relationship to a nodal point. We also explored the connection of this phenomenon to floating signifiers—terms or symbols lacking a fixed, stable meaning, thereby inviting diverse interpretations. This connection highlights the ambiguity or openness inherent in nodal points.

Furthermore, we focused on the subject’s role in the discourse. Subject positioning can be understood as a social construction in which people as subjects position themselves and others, in this case during the interview, as well as how others position them in discourses. Subject positioning in discourses illustrates the generative nature of discursive power, not only as a repressive force but also as a facilitator of agency and productivity. This intricate process involves adaptation to diverse discourses (Butler, 1995).

Discourse analysis also examines subjectivity via interpellation, in which specific subject positions are ascribed and attributed certain characteristics and traits embedded in the discourse (Althusser, 1976; Butler, 1993; Fairclough, 1992).

### Focus Groups

Drawing from the principles of social constructivism, focus group methodology hinges on participant interaction to clarify viewpoints, stimulate discussions and explore emerging issues (Barbour, 2018; Ivanoff & Hultberg, 2006). This approach stands out from other interactive methods by its deliberate emphasis on utilizing group interaction to generate data on a research-selected topic (Krueger, 2000; Morgan, 1997). Focus group studies are particularly apt for the study of attitudes and values (Barbour, 2018).

Communication among participants plays a pivotal role in shaping the outcomes, as the group process encourages them not only to express their thoughts but also to elucidate the underlying reasoning (Kitzinger, 1994). The focus on group interaction enables the exploration of diverse viewpoints and opinions, thereby facilitating comprehensive data collection (Ivanoff, 2002; Morgan, 1997).

### Study Context

This study was conducted in a city in Sweden. The Swedish healthcare system provides universal healthcare coverage for all residents and is publicly financed along with a minimal patient fee (Janlöv et al., 2023). It is divided into three levels of administration: state, regions and municipalities. Specialized palliative care is organized by regions and municipalities. This study involved professionals from both levels of administration.

### Participants

Five focus groups (5–7 participants in each group) were conducted, with a total of 27 palliative care professionals participating, active in specialized palliative home care. Smaller focus groups (up to six participants) have been found preferable to larger groups, as this allows for greater opportunity for dynamic discussions and ample opportunity for all participants to express their views (Ivanoff, 2002). Four of the groups had participants from the region, and one from the municipalities. Efforts were made to ensure homogeneity among the participants by emphasizing shared experiences in specialized palliative care. In each group, heterogeneity in age, years in palliative care, profession and workplace was striven for to capture a diversity of experiences and to broaden the discussions (Table 1) (Barbour, 2018; Ivanoff & Hultberg, 2006; Kitzinger, 1994).

**Table 1:** Participant characteristics (*n* = 27)

Registered nurses	14
Physicians/consultants	4
Health social workers	5
Various professions*	4
Gender	
Female	25
Male	2
Age, mean (range)	46 (26–68)
Professional experience in palliative care, years, mean (range)	7 (1–40)

^*^Various professions include assistant nurses, physiotherapists, occupational therapists. Due to ethical considerations, professions of those with few representatives are withheld to avoid potential identification

### Procedure

Invitations to participate were extended to participants through the management leader who then provided them with written details about the study’s objectives and the expected level of participation. Those interested were scheduled for an upcoming focus group at their workplace. The focus groups were conducted during the participants’ working hours in March and April 2023. They lasted between 68 and 80 min.

The focus groups started with a presentation round encompassing name, profession, and years in palliative care. The presentation round was followed by key questions: (1) What challenges do you face in relation to issues related to religion and spirituality? (2) How do you reason (based on your professional role/based on your experiences) regarding patients’ right to spiritual care and healthcare’s responsibilities regarding patients’ spiritual and existential needs? (3) Based on what we have discussed, how would you wish healthcare’s handling of religion/spirituality to be?

The first author (EL) moderated all five focus groups, LD observed two, DE and JÖ observed one each. One focus group was held without any observer present. The moderator’s role included encouraging interaction and ensuring that all participants had a chance to express themselves. Efforts were made to involve every member in the discussion. Drawing from Ivanoff (2002), the group leader avoided answering questions from group members, preferring to redirect them to the group for discussion.

Active listening was prioritized, with the leader focusing on understanding the conversation’s essence and direction. Follow-up questions, like “Could you elaborate on…?” were used to deepen the discussion. Observers focused on documenting nonverbal cues and interruptions in their field notes, which were discussed in direct connection to the focus groups. The discussions were recorded and transcribed verbatim.

### Ethical Consideration

The present study was ethically approved by the Swedish Ethical Review Authority (Dnr 2019–05116). Before the focus groups, all participants were informed about the objectives of the study and were reassured about the confidentiality of their personal information. Informed consent was obtained from all participants.

## Results

### Understanding Religion, Spirituality and Spiritual Care

There was agreement among all participants that spiritual care, caring for the patients’ spiritual well-being, was part of palliative care philosophy, especially in specialized palliative care. A few referred to the theoretical concept of “total pain,” a concept coined by Dame Cicely Saunders, the founder of the hospice movement. Some also referred to how caring for all aspects of pain lies at the core of palliative care.

However, the concept of “spiritual care” was surrounded by confusion and was often discussed in relation to “existential care.” A majority wished for definitions and distinction between the concepts of spiritual and existential, and there was a recurring need to separate them from each other. One physician explained:

I don’t really like the expression “spiritual care”; I don’t know what we’re talking about. Spiritual and existential [care], those are different things. What are we talking about? […] I have a hard time understanding what you mean, what you want, what you’re thinking about, because then I think it [spirituality] has something to do with not being religious but is still about God or something. So, for me, it’s a difficult concept; I don’t understand it at all. Existential, that’s something else (PH1).

This physician made a clear distinction between spiritual and existential care, and specifically asked for clarity and definitions before continuing the discussion. Here, spiritual care was connected to “God or something,” but still not religious. “Something” could be seen as connected to God or a higher power. However, to this physician, being spiritual was not the same as being religious. A contrasting view on definitions was presented by another physician:

For me, it’s not a problem. I find them so closely intertwined that I often don’t distinguish between the two [existential and spiritual]. I know they are two different concepts, but in practice, it’s rarely purely an existential issue because they are so heavily influenced by culture, religion, and spirituality. Personally, I seldom try to separate them because I often find it more helpful just to get it out, regardless of the labels, and then think about how to approach it or, from a scientific perspective, how to address it (PH2).

Here, the physician recognized that in theory the concepts are separate, but in healthcare practice, intertwined and hard to separate, and pointed out that for her, there was no need to separate them. Regardless of existential or spiritual concern, the medical treatment would be the same. It has also been pointed out that spiritual and existential concerns should be addressed from a “scientific” perspective.

The understanding of spiritual care was mainly connected to the concepts of spirituality and religion. Spirituality and religion were discussed as intimate, private matters and spheres that required great caution to enter. One healthcare counselor explained:

How do you open up to something that is still very private, especially when I might not have the knowledge of what it entails and perhaps do not belong to that religion? There are many factors to take in; it’s private, and I also lack knowledge and do not belong to that religion (HCC1).

There are three key concerns related to spiritual care mentioned here: (1) religion and spirituality as private; (2) lack of knowledge; and (3) not belonging to *that* religion. The degree of privacy in relation to spirituality and religion seemed to be connected to knowledge and whether you could personally relate to the patient’s belief system or not.

A recurring argument was that it was easier to approach religiosity if you had knowledge of or belonged to that tradition. Implicitly, there was an assumption that religion was less private if religion was material and physically manifested (through attributes and acts such as clothing, jewelry, a crucifix, a veil, prayer or food preferences), or if you had shared beliefs. Very few participants asked patients about their religiosity, unless it was visible or if the patient mentioned God. Participants also articulated “fear of getting information that you might not know how to handle” which led to avoidance.

There was a general assumption that if religion was not visible but still of importance to a patient, the patient would orally inform healthcare professionals of their beliefs. Otherwise, healthcare professionals did not have to attend to it. This assumption puts the responsibility on the patient. This can be seen as a way of legitimizing why it was acceptable *not* to ask about the patient’s religiosity.

In this manner, visible religion was considered less private. The assumption that spirituality and religion were something that could be “seen” was articulated and prominent throughout the focus groups.

One registered nurse explained: “When you see someone who is Hindu, you often see that dot (on the forehead, called a *bindi*), and then you understand that there are many ritual aspects surrounding it. In those cases, you almost have to ask; if not, you might run into many different surprises” (RN1). In this view, artifacts and symbols not only reveal, but also actively tell healthcare professionals what religion or tradition a person belongs to.

A general assumption was that the experience of dying was “less painful,” “easier” and “calmer” for patients who had a belief to lean on. Religion and “belief” were seen as helpful in coping with impending death. There was a common understanding that people become more religious at the end of life. Faith and religiosity were sometimes discussed as a “last resort,” when patients had nothing left to turn to. One other healthcare professional explained:

Believers had a completely different calmness when facing death because they knew that paradise awaits on the other side. Meanwhile, those without faith were more anxious about what happens. I believe they [believers] find a sense of peace in their faith somehow, and that’s truly remarkable if someone can do that. Security (OHCP1).

The focus here was on the “afterlife,” and on the main challenge connected to dying which was whether there was an afterlife or not. “Paradise” was described as something that awaits all people of faith, regardless of religion, which made passing away easier. Faith provided peace and security for dying persons (i.e., death without anxiety). There was an implicit statement here that a calm death is a desirable death and that it was harder to approach spirituality and religion (i.e., carry out spiritual care,) for those without any belief.

### Negotiating “Us” and “Them”

In the focus groups, the participants explicitly positioned themselves as Swedish and secular, and many of the participants pointed out that they were not religious. Even those who identified themselves as Christians pointed out that “I am not religious *like that,”* referring to “strong beliefs.” Included in the concept of being “secular” was being nonreligious. This was articulated in one of the conversations:

RN2: I can find it challenging in general to deal with spirituality or religiosity in people, especially for patients or their family members where it holds significant importance in their lives. It’s crucial and sincere for them, whereas I, being somewhat atheist or at least nonreligious, have a different perspective. I need to navigate the fact that they have a different reality and a different expectation toward death. Handling this requires a level of humility, regardless of their specific religion. It can be a challenge to be present in that space without fully embracing it, but at the same time, I cannot dismiss their beliefs.

RN3: I would say that most people we encounter are secular and may not have any prominent faith. We may not encounter religion as often as in other countries with a stronger [religious] tradition.

OHCP2: There might have been a shift in faith […] We are more and more secular or secularized.

RN4: Yes, Sweden is quite secular.

These palliative care professionals’ positioning as Swedish, secular and atheist or less religious was evident in this conversation. Sweden being secular was explained as a reason for why they might not encounter religion as often as in “other” countries that are “more religious.”

“Religion” in this instance was clearly connected to organized religions such as the world religions and religiosity. There are implicit statements that religion belongs to “them” (other countries, less secular, non-Swedish) and not “us” (Swedish, secular, atheists). However, it was pointed out that secular people have “less prominent” faith which can be interpreted as acknowledging the presence of less “visible” forms of religion and faith. The positioning of “us” and “them,” included situating Sweden in the “west” and as embracing “western ideas” and perspectives. One healthcare counselor reasoned about her own preconception:

If they ask a question related to their religiosity or cultural aspects […] how does it align with our Western perspective. I assumed now that it’s not a western perspective […] as in all other aspects, I might say: “I’m not sure, but I’d gladly find out.” It is kind of our thing [finding out] (HCC2).

The healthcare counselor was aware of healthcare professionals’ preconceived notions about religion and culture not belonging to “western perspectives.” However, she confirmed that questions about religion and culture were not treated differently from other questions. Part of healthcare professionals’ professional responsibility, regardless of issue, was “finding out” things.

Positioning as “us” and “them” occurred frequently in relation to “truth-telling.” Not giving full information to patients about their situation was seen as an ethical conflict and was mostly discussed as a “consequence” of belief or religion. It was discussed mainly in relation to Islam and Muslims. One nurse explained:

It can be in conflict with our mission to be clear and provide information and involve the patient because sometimes we receive indications from relatives in a religious context that we shouldn’t shatter someone’s hopes by being too explicit with information. I can perceive this as a bit of a challenge; we don’t want to disrupt anything, but we still have an obligation to the patient, who traditionally should be autonomous and have their say about the care to be provided and what should happen at the end. Sometimes, I can find this a bit problematic (RN4).

This was a recurrent pattern in how many participants reasoned. In this case, she positioned herself as a professional and as a provider of information (i.e., “the truth”). It was pointed out that in her profession (nursing), the mission is to include the patient and to provide information. The patient is acknowledged as “traditionally” autonomous, referring to the principle of autonomy in health care.

The issue of truth telling was described as especially challenging when children were involved. Not informing young children of a parent’s illness and prognosis was seen as conflicting with healthcare ethics. Again, HCC1 explains:

One challenge arises when dealing with a family with children who, due to their religion and cultural background, may not want to disclose the illness or impending death of a parent to the child. It poses a challenge for us, as the research we have access to in the Western world often suggests that it’s beneficial for children to be informed as much as possible about what is happening. This can create a conflict at times. It can be a challenge for the team to balance respecting the religion or culture while also considering the best interests of the children.

Again, references were made to the “west,” marking a western hegemony. Emphasis was put on science and research conducted in the “west,” assuming that outside the “west” there might not be “access” to the same knowledge. It was implied that religion and culture did not align with “western” views on the “children’s best.” The conflict, according to the healthcare counselor, was in both respecting religion and culture while at the same time considering what was best for the child. A physician shared a similar story:

I tried in my eagerness to bring in the perspective of the children, you know, and it was very clear that it was her culture, if I understood correctly, and her belief that this [her illness] was not something to be shared with the family in any way, this is how it’s done, and I shouldn’t come in as a doctor and tell her how to deal with this. Whether it’s religion, spirituality, or just culture, I don’t know how to distinguish between them, but it becomes very difficult to be a doctor (PH1).

**Fig. 1:**
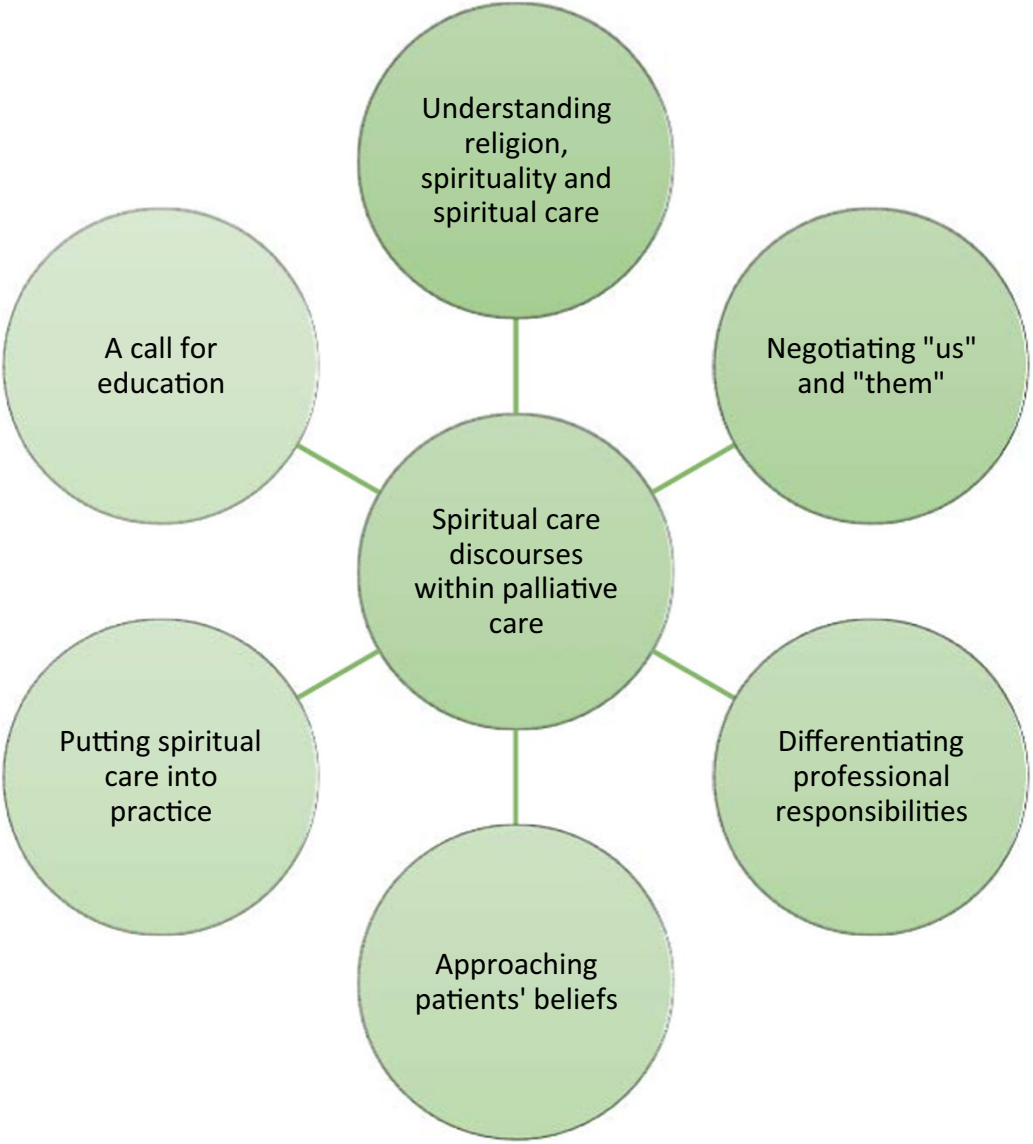
Overview of the results

Here, the physician’s view of the children’s perspectives did not align with what she understood as the patient’s culture and belief. The patient’s view was assumed as conflicting with the “western” view, and this caused a dilemma. We found two different perspectives here: the patient who believed the physician was there simply for medical reasons that should be directed toward her as patient, and the physician’s conflicting view that had a wider perspective and saw caring for the patient’s family and informing them about children’s perspectives as included in her mission (Fig. 1).

### Professional Responsibilities

All professions agreed that, to some extent, it was their professional responsibility to address and attend to their patients’ spiritual and religious needs. It was argued that spiritual care was a shared responsibility and was not bound to a single profession. However, this did not necessarily mean that every healthcare professional “can or should be the one to provide it.” There was a separation between “can or should,” implicitly stating that sometimes professionals *should not* engage in their patients’ spiritual needs. Sometimes there are other professions that are more fit for the task, like a religious representative or healthcare counselor. One physician emphasized the team:

I believe that in the kind of work we’re involved in, it’s important that someone on the team introduces it. It’s absolutely inconsequential whether it’s the doctor, occupational therapist, healthcare counselor, nurse […] – it makes no difference. Sometimes, as a doctor, you may be seen as a more trustworthy figure, so maybe it’s the doctor who can do it. In some cases, it’s the opposite, and it doesn’t open up when it’s the doctor (PH3).

Here, the physician also pointed out that the “kind of work” (palliative care) they are involved in is a specific approach, and that in palliative care it is important that spiritual needs are met. That spiritual questioning increases with an impending death is implied here. It was also pointed out how physicians might be seen as “more trustworthy.” However, despite general agreement on shared responsibility, there were those who opposed this idea:

I believe that we [physicians] are proficient in palliative care, so to speak, and we have healthcare counselor who are skilled in their role, and we have psychologists who excel in their domain. Then we have deacons, priests, and chaplains, among others, who are experts in their respective areas. I personally think I should become better at encouraging patients to make such connections because I believe it could be immensely helpful (PH4).

Here, a differentiation was indicated between the professions. By claiming that physicians should be better at linking patients with the right profession, the physician implies that spiritual care is not a physician’s field of expertise. By postulating that physicians are proficient in palliative care, while healthcare counselors, psychologists, deacons, priests and chaplains all have their specific fields of expertise and responsibility, this physician equated palliative care with palliative medicine. By acknowledging each profession’s field of expertise, physicians’ expertise in the strictly medical dimension of palliative care is highlighted.

Furthermore, in relation to professional responsibility, nurses argued that *spiritual care* should be provided by other professions (i.e., religious representatives), and that the nurse’s role was to facilitate this, not to provide it themselves. Spiritual care was connected to religious questionings and should thus be referred to representatives from the religious traditions**.**

### Approaching Patients’ Beliefs

Highlighted in all focus groups, among all professions, was an insecurity in not being able to engage correctly in conversation regarding the patients’ spiritual and religious dimensions. The idea of spirituality and religion as private, intimate and personal, made it a complex topic to engage in and ask about. A lot of participants emphasized the importance of asking about patients’ beliefs. However, this was often in relation to practicalities that arose when the patient had passed away.

Certain religions were said to have specific needs and rituals that influenced end-of-life decisions and the actual handling of the dead body. One nurse explained:

I usually ask early on if they are religious or belong to any religion […] If they are Muslims, or Christian Catholic, there are practical considerations that can come with death. For example if you are a Muslim, you must be buried within three or four days (RN5).

Swift burial in Islam was a well-known aspect that was raised by several participants. One of the nurses explained:

What’s absolutely the clearest to me is when a person passes away, and there’s a rush to start the ritual washing, and an imam needs to come, and the whole process must happen within 12 or 24 hours, so it’s very urgent. Often, a person from the family who hasn’t been present very much during the patient’s life steps forward to take responsibility for initiating this process, usually a son or a male family member. We need to issue a death certificate quickly for this, or else the ritual washing won’t start. It becomes so urgent, and that’s what it’s all about. I usually want to say, when someone passes away, take your time; there’s no hurry (RN6).

Several key issues are mentioned here that are related to religion: a calm death, washing (*ghusl*) and a swift burial. Ritual washing and the process surrounding it was seen as stressful and did not align with the view shared by most healthcare professionals of “a calm death,” without stress. Throughout the focus groups, there was significant focus on the impact of religion after death.

In contrast to this, one nurse explained how religion also holds significance for patients before they pass away, emphasizing that professionals should also pay attention to this before patients pass away:

I also think it’s good to know such things [spirituality and religion] even before they pass away, especially things that are important to them […] A patient I visited, the only thing she needed help with and talked about was that she wanted the washbasin somehow lowered or fixed so that she could wash her feet. It was really the most important thing for her, and it took us a while to realize that it was because she needed to pray. She couldn’t pray without washing her feet (RN2).

Here, practicalities in relation to religion were emphasized, and how religion was connected to the patient’s well-being was also pointed out. Religion was also seen to have an impact on medical treatment. One nurse described this:

Some individuals, such as Jehovah’s Witnesses, I believe, are not allowed to accept blood transfusions. There we face a challenge because we know that a blood transfusion would benefit the person, but their faith dictates that they must refuse it. In such situations, we find ourselves in a dilemma, but we should simply respect their decision, I think. We can’t insist on something if they have firmly decided that it goes against their beliefs or isn’t something their faith allows (RN1).

Here, the emphasis was on the medical treatment and how it would benefit the patient. What was “beneficial” here was based on medicine and the priority was to tend to the physical body and its needs. In this example, religion dictated what the religious person could or could not do, implying that the beliefs of Jehovah’s Witnesses are rigid and inflexible.

When patients choose to put their spiritual well-being (not receiving blood) before their medical needs, it causes confusion and ethical stress for palliative care professionals. Not accepting blood transfusions was compared with patients refusing intravenous nutrition or refusing to eat. Participants agreed that these situations were challenging but that they “of course” had to accept them and make the best of them.

Willingness to do what was best for patients prevailed, with efforts to see possibilities instead of obstacles. However, sometimes professionals failed to apply this approach. Several participants shared a story of a young Muslim patient who decided to travel to Mecca (*hajj*) before he died. They were extremely worried that the patient would not survive the trip and the physician urged him not to go.

Despite urging him not to go, the young man traveled to Mecca. And “as expected,” that had significant medical consequences. Despite knowing that this man was a Muslim, they advised him not to go, leading to regret and anxiety for team members. A physician explained:

I really made a mistake when I met this man who was going to Saudi Arabia. He said, “I want to go to Saudi Arabia,” and we had this really catastrophic idea. Then I told him that, unfortunately, the airlines […] if someone is seriously ill and at risk of dying on board, they don’t want to make an emergency landing. So, I’m sorry, I said. He was upset, and then he just left. But I should have realized that he’s a Muslim and he wants to go to Saudi Arabia to visit Mecca, which is almost essential for a Muslim […] I should have thought a bit further and said: “Let’s plan a well-organized trip to Saudi Arabia” (PH4).

The physician admitted that they were too focused on the medical issues, and that the patient’s spiritual andreligious needs were overlooked and neglected.

### Putting Spiritual Care into Practice

Throughout the focus groups, participants emphasized that to be able to attend to the patients’ spiritual and religious needs, it was necessary above all to build a relationship with them. Since spirituality and religion were often seen as private, one essential requirement for spiritual care was to establish a relationship in which the patient felt comfortable and safe. Building a relationship also required time, which most professionals felt that they had. They pointed out that they therefore had what is required for the engagement in conversations on spirituality and religion.

In the encounters they needed to be able to “sit in silence,” “set aside their own values,” and first and foremost have the ability to “listen.” Conversations on spiritual care centered on “providing support” and “being present.”

RN7: I don’t think people often seek affirmation of their faith, but rather the comfort of being able to talk about it. Many people find security in being able to share their beliefs. It’s somehow reassuring for them to explain and talk about their feelings, thoughts, and opinions, and we just need to listen, not necessarily agree or affirm in any way.

RN8: We must be prepared to receive that and have the courage to listen.

Here, spiritual care was considered a courageous act. “Courage” can be understood as necessary for standing still and being present even if it is uncomfortable and one has no answers. Participants stressed that nobody is expected to have any answers, just being there is enough. “Being there” and “listening” were considered spiritual care acts in themselves.

“Being present” also works as a catalyst; when a professional is present with a patient, spiritual and existential conversations can emerge. In these conversations, the emphasis is on sharing views with patients as “fellow human beings,” not as specialists.

Lack of personal belief might constitute a hinder in conversations with patients, as this weakened professionals’ understanding. Not having any personal faith was sometimes considered a bit “shameful” when caring for persons at the end of life. Believing that there is “nothing more” waiting after death was seen as taking away patients’ hope.

At the same time, most professionals were uncomfortable about introducing their own beliefs into conversations. Some were comfortable talking about spiritual issues as long as questions were directed toward the patient but experienced discomfort when questions about belief and faith were turned in their direction. One nurse explained: “I find it difficult to receive that question. It’s not what I want to focus on in the conversation in any way” (RN9).

Receiving questions had the potential of blurring patient-provider boundaries, and professionals wanted to keep conversations one-way. One method for handling this was to “bounce back” any questions, and to ask questions like “What do you personally believe? What are your thoughts?”.

Several participants raised the point that it would be good to formalize questions about spirituality and religion. As one other healthcare professional explained: “It’s tough, but you just have to start asking questions; ‘have your existential needs been met?’ You just really say it out loud to get rid of it” (OHCP3). In this example, an active approach was promoted. Asking about patients’ beliefs may be one of those questions professionals just tick off, as one nurse explained:

The “do you have any belief?”, is still a fairly practical question, if you put it that way, do you have it or do you not, and the patient can tell you. Alternatively, what has kept you going during difficult times in your life? I believe that one’s life story is also a form of spirituality for oneself, to be able to tell someone how life has been (RN10).

Here, the question of belief could be seen as a practical question among others, and at the same time work as a catalyst to give the patient an opportunity to open up. The idea was also raised that listening to a patient’s narrative could be seen as a spiritual care act, that there were spiritual dimensions in telling life stories.

However, there was disagreement about whether these questions should be formalized or not. Some argued that it is too private to ask about at a first meeting. Furthermore, all the participants argued that there were different ways of engaging in or asking about spirituality and religion. There was no “one size fits all” solution.

Participants showed minimal participation in patients’ religiosity and had concerns about crossing boundaries. However, instances of engagement included singing or praying with patients, holding hands during prayer, providing bibles, washing feet before prayer and involvement in funeral-related activities. Attending or assisting in the planning of funerals emerged as the most common form of spiritual care. In this way, spiritual care extended beyond the patient’s life, encompassing the period before death, during death and after death.

### A Call for Education

Recurring in the focus groups was a call for more education on spirituality, religion and culture, specifically more “general knowledge.” To some extent, there was an essentializing understanding of religion, in which all persons belonging to a certain religion shared beliefs and rituals regarding death. However, the assumption of universal knowledge about religions and cultures was challenged by those arguing for diversity within religions and that “one size doesn’t fit all.”

Despite this, most participants asked for education in “general knowledge” about the major religious traditions. They all agreed that education and knowledge on the topic would benefit them in their encounters with patients. A healthcare counselor explained: “There is a desire to always respond with respect, and it’s challenging when one doesn’t always have enough knowledge” (HCC3).

## Discussion

Through the use of discourse analysis, we were able to uncover implicit understandings of “spiritual care” among palliative care professionals. This study explored the underlying narratives and themes shaping the discourse. Our analysis reveals a nuanced exploration of the discourse, with particular emphasis on the pivotal nodal points of “spirituality” and “religion.” This discussion critically examines the implications, nuances and broader patterns that emerged, shedding light on the multifaceted dimensions of the discourse surrounding spiritual care.

Frequent in the focus groups was a need to separate the concepts of “spiritual” and “existential.” “Spiritual” was perceived as vague and hard to grasp, challenging the idea of responsibilities. The need for distinction between “spiritual” and “existential” may be seen as a reflection of contemporary Swedish healthcare and healthcare research literature, in which the concept of “spirituality” has a religious connotation and in which the discourse on “existential care” is more prominent than that on “spiritual care.”

The term “existential” frequently appears in Swedish white papers and official documents, diverging from the prevalent international research in nursing that typically embraces the consensus-based concept of spirituality (Nygaard et al., 2022). “Existential” is sometimes promoted as serving better than “spiritual” as it is perceived as more open to the secular dimensions. In the Swedish context, there is a tendency to speak in terms of existential questions rather than spiritual care (Karlsson et al., 2014; Nygaard et al., 2022; Willander, 2023) and to mix them with psychosocial needs. This may further situate religion and spirituality as factors unfamiliar to healthcare professionals (Dellenborg & Enstedt, 2023a, b).

Even though existential and spiritual care may be seen as overlapping in some regards, it is evident in both previous research and the focus group discussions that there is an urge to use these concepts as distinct from each other, since each is seen to have its advantages and since they are not interchangeable. However, while making such a distinction, there is also a problematic tendency to essentialize the two concepts. For instance, Hvidt et al. (2022) argue that “‘spirituality’ has an inner energy and refers to the intrinsic source of one’s being and motivation” (p. 3297). Evident here is an essentialist view on spirituality, which, as we concluded in previous research, dominates the spiritual care discourse (Lundberg et al., 2024). This essentializing view on spirituality and religion might lead to lower care quality, as it may overlook individual variations, thus leading to stereotyping and prejudice.

As noted above, the decline in religious activity and values does not necessarily equate with an overall decrease in belief. Throughout the focus groups we saw how participants positioned themselves as spiritual but not religious. The shift away from “religion” can be explained in the context of prevalent ideas and values associated with secularism. Instead of adhering to religious practices, divergence from religion leads to an inclination toward spirituality.

Spirituality can be said to place importance on the authority of one’s inner, subjective experiences and typically adopts a universal or “holistic” orientation (Vincett & Woodhead, 2016), whereas religion is characterized by its allegiance to tradition, doctrine and established authority. Heelas and Woodhead (2005) describe how people turn to *subjective-life* religion, in which the authority is in the individual, a form of religion that can be referred to as “spiritual but not religious” (SBNR) (Parsons & Ebscohost, 2018).

Sociologist Thomas Luckmann (1967) used the term “invisible religion” to describe how various social expressions of religion are transforming in society, not necessarily diminishing. Another way to discuss this is through Bailey’s (1997) concept of “implicit religion.” Invisible religion has been set in contrast to more visible, ecclesiastically-orientated religion (i.e., “visible religion”) (Enstedt & Plank, 2023a, 2023b).

Discussions in the focus groups mainly circulated around visible religion for which substantial definitions dominated. Religion was described in its visible content: artifacts, symbols and rituals. Visible religion was described as a help in approaching patients’ religiosity. At the same time, this approach poses the risk of not acknowledging less visible forms of religion, thus not approaching the religious or spiritual dimensions of a patient and therefore failing in providing spiritual care for all (if needed).

In relation to this, invisible religion was seen as private, and visible religion less private. The private and the public were clearly articulated as being in opposition to one another, revealing a dichotomous model. This dichotomy was recurrent in the focus groups as a positioning of “us and them,” with “us” being Swedish, secular and informed by science, and “them” being traditional, religious, non-Swedish and non-western.

Embedded in the idea of the secular is the view of secular people as a religious, modern and believers in science. This can be seen in the light of “othering,” a concept developed and applied in feminist theory, dating back to de Beauvoir’s (1949) work on the construction of men and women, and further elaborated by postcolonial theorists such as Said (1978) and Loomba (2015). Othering is a procedure that identifies individuals perceived as different from oneself or the prevailing norm and also a process through which people construct their own identities in reference to others. Terms like “secular,” “religious” and “western” were frequent markers that signalized othering.

Religious and cultural definitions are often used to explain differences and categorize groups or individuals as “other.” Stereotypical views on healthcare practices in cultural and religious groups may on the other hand oversimplify behaviors, values and beliefs, overlooking individuality and diversity (Johnson et al., 2004). In this study, we found a call for “general descriptions” of cultures and religions, and while these might guide healthcare professionals working with diverse groups, they can also contribute to the marginalization of these groups as “outsiders.” Despite shared belief that religions cannot be generalized, generalizations were repeatedly revealed.

Othering language appeared foremost in descriptions of situations that healthcare professionals found difficult, and it became especially frequent in relation to “truth telling,” referring to ethical principles and practices of providing accurate and honest information to patients and their families. The positioning of “us” and “them” was clearly highlighted with such references as “secular,” “science” and “non-religious.” Truth telling, a cardinal rule in medicine (Gold, 2004), is, however, not a globally shared moral stance in medicine (de Pentheny O'Kelly et al., 2011).

Not being able to inform patients was seen as creating ethical conflict and stress for the palliative care professionals. In a review, Tuckett (2004) concludes that it is the principle of patient autonomy and the likelihood of bodily and inter-relational harm that argues both for and against truth telling in clinical practice.

Furthermore, the notion of truth telling and especially children’s rights can be described using the concept of clash of values developed by Linda Woodhead, a sociologist of religion. The antagonism between different positions in what is best for children can be seen as being based on the same values. What sets them apart is that different meanings are attributed to these values in the respective contexts (Dellenborg, 2020). From this perspective, the Swedish values of children’s rights are connected to “a sacred narrative of (European) secular progress” (Woodhead, 2009, p 12). Other views of children’s rights not only become a threat to this “sacred narrative,” but also become a threat to the foundations of western civilization.

In the focus groups, we saw a call for heightened educational focus on spirituality and religion. The call is for comprehensive understanding encompassing both “general knowledge” and “basic features” of religions, and also the elucidation of various traditions and their associated rituals. Evident in this is a substantive definition and a *sui generis* understanding of religions (McCutcheon, 1997), thereby rendering religions as self-contained entities. The utilization of the term “basic features” in conjunction with the conceptualization of fundamental ideas and rituals, exposes an inherently essentialist perspective on religion.

The dominance of essentializing views on religion in palliative care research has been discussed in Lundberg et al. (2024). The comprehension of religions, as delineated in the focus groups, proves problematic, depicting “religions” in fundamentally misleading manners rooted in a *sui generis* framework derived from the World Religious Paradigm (WRP) (Cotter & Robertson, 2016). The WRP represents the intersection of knowledge and according to Foucault (2001) can be called a particular “episteme”—knowledge that has been internalized and will appear and claim to be universal, eternal and natural.

Critics argue that the WRP is rooted in western concepts of religion, potentially resulting in oversimplification and an inability to acknowledge religious expressions that exist outside this framework (Enstedt, 2020). Critiques of this essentialized perspective are prominent in the works of Cotter and Robertson (2016) and Horii (2018) as well as in the field of critical religion, which strongly challenges the notion of fixed and inflexible religious identities.

Education on religion, spirituality and spiritual care can be discussed through the concept of religious literacy, a field that has attracted attention recently in health care and elsewhere (Dinham, 2018; Enstedt & Dellenborg, 2024; Pentaris, 2019; Walker et al., 2021). Suggestions have been put forward regarding how to increase religious literacy in society, education and health care (e.g., Chan & Sitek, 2021; Moore, 2014; Pentaris, 2019). Our understanding of religious literacy is in line with Dinham and Francis (2016) who describe it as the knowledge and understanding of religion and belief, as well as the skills and abilities required to engage with them. This includes understanding that religions are internally differentiated and contextual (Dinham, 2018).

In previous research (Lundberg et al., 2024), we concluded that palliative care research contributed to the moral formation of healthcare professionals, interpellating and ascribing qualities that healthcare professionals need to meet patients’ spiritual needs effectively. Lundberg et al. (2024) concluded that qualities such as being an empathic listener, being “present” and courageous were highlighted. The same qualities were found in this study and were described as a significant part of spiritual care. Similar characteristics have also been described by Feldthusen et al. (2022) regarding person-centered care and other types of “centredness” in health care.

Our study also showed that healthcare professionals desired spiritual care to be a one-way conversation. This can be seen as a contrast in relation to their focus on building relationships as a central aspect of spiritual care. Schuster (2006) highlights how necessary healthcare professionals’ will and courage are to meet a vulnerable other with their own life experience as a base for resonance. Spiritual care centered on listening and “being present” which involves dialog where the healthcare provider uses their personal experiences in life to be present; however, such conversations may have various underpinnings and shapes (Cf. Öhlén & Friberg, 2023).

## Strengths and Limitations

Discourse analysis offers a systematic and nuanced approach to understanding the social construction of meaning in conversations. By applying this method to focus groups, we gain comprehensive understanding of how participants collectively shape and negotiate meaning in a given context. The strength thus lies in the ability of discourse analysis to uncover underlying power dynamics, cultural nuances and implicit assumptions that may not be immediately apparent. Moreover, this method allows for a detailed exploration of language use, contributing to a richer interpretation of participants’ perspectives.

However, we acknowledge certain limitations inherent in this study. Firstly, the scope of our investigation was intentionally directed toward challenges associated with spiritual care in palliative care. The formulation of questions primarily emphasizing difficulties might have influenced participants’ responses, potentially leading to an underrepresentation of positive aspects or successful spiritual care practices.

Furthermore, we acknowledge the potential for subjective interpretation and the contextual specificity of findings which may limit the transferability of findings to other cultural or healthcare settings. To mitigate this, we actively worked with our pre-understanding by critically examining our assumptions, biases and prior knowledge, and consciously challenging them throughout the research process.

## Conclusions

Despite palliative care professionals’ intentions of promoting equality, language can contribute to misinformed notions about religions and spiritualities, leading to unintentional othering practices. The view of belonging to a secular society, including secularism’s associated values and ideas, hindered palliative care professionals in their encounters with religion. However, participants positioned themselves as spiritual but not religious and the understanding of spiritual care centered around “being present” and the practice of active “listening.”

Based on this study, we found a static understanding of religion which illuminated the need for religious literacy and a deeper understanding of diverse religions and spiritualities. In addition to identifying visible forms of religion, the results of this study also indicate a need among healthcare professionals to discern, understand and relate to the non-visible forms of religion that they encounter in their everyday practice in palliative care.
