# Correction to: Navigating Complexity: Spiritual Care Discourses Among Swedish Palliative Care Professionals

**DOI:** 10.1007/s10943-024-02133-1

**Published:** 2024-09-19

**Authors:** Emma Lundberg, Anneli Ozanne, Lisen Dellenborg, Joakim Öhlén, Daniel Enstedt

**Affiliations:** 1 Institute of Health and Care Sciences, Sahlgrenska Academy, University of Gothenburg, P.O. Box 457, 413 46 Gothenburg, Sweden; 2 Department of Neurology, Sahlgrenska University Hospital, Blå Stråket 7, 413 46 Göteborg, Sweden; 3 Centre for Person‑Centred Care (GPCC), University of Gothenburg, Arvid Wallgrens Backe, 413 46 Gothenburg, Sweden; 4 Palliative Centre, Sahlgrenska University Hospital Region Västra Götaland, Lilla Kapplandsgatan 7C, 421 37 Västra Frölunda, Sweden; 5 Department of Literature, History of Ideas, and Religion, University of Gothenburg, Renströmsgatan 6, 412 55 Gothenburg, Sweden

**Correction to: Journal of Religion and Health** 10.1007/s10943-024-02106-4

In this article the author name “Daniel Enstedt” was incorrectly written as “Daniel Ensted”.

The original article has been corrected.

